# The Role of the Keratinized Mucosa in Peri‐Implant Diseases Onset and Brushing Discomfort: A 10‐Year Follow‐Up

**DOI:** 10.1111/clr.70123

**Published:** 2026-03-29

**Authors:** Fernanda Angelio da Costa Deller, Jeniffer Perussolo, André Barbisan de Souza, Ricardo Puziol de Oliveira, Flávia Matarazzo, Maurício G Araújo

**Affiliations:** ^1^ Department of Dentistry State University of Maringá Paraná Brazil; ^2^ Centre for Oral Clinical Research, Institute of Dentistry, Faculty of Medicine and Dentistry Queen Mary University of London UK; ^3^ Department of Periodontology Nova Southeastern University Davie Florida USA; ^4^ Department of Statistics São Paulo State University Presidente Prudente Brazil

**Keywords:** dental implants, dental plaque, gingival recession, inflammation, oral hygiene, oral mucosa

## Abstract

**Objectives:**

To evaluate the effect of narrow (< 2 mm) and wide (≥ 2 mm) keratinized mucosa (KM) over 10 years on peri‐implant diagnosis and brushing discomfort.

**Materials and Methods:**

Eighty patients were initially evaluated. Demographic data, modified plaque index (mPI)/plaque index (PI), probing depth (PD), clinical attachment level (CAL), mucosal recession (REC), bleeding on probing (BoP), marginal bone level (MBL), brushing discomfort (BD), and peri‐implant diagnosis were assessed. Implants were then divided into two groups according to the width of KM (narrow group: NG < 2 mm and wide group: WG ≥ 2 mm). Patients who returned after 4 years (T4) were invited to participate in the 10‐year reassessment (T10). The same parameters were reassessed, and statistical analysis was performed.

**Results:**

Thirty patients and 116 implants were included in this 10‐year follow‐up study. Although at T10 the prevalence of peri‐implant diseases was not influenced by the KM width, the implants in the WG had 84% lower odds of being diagnosed with peri‐implant diseases compared to those in the NG. Furthermore, the NG had higher PI, mPI, BoP, and REC than the WG. In addition, significant MBL was observed in the NG over 10 years. Both groups reduced BD, with no significant difference between them.

**Conclusion:**

A narrow KM favors plaque accumulation, mucosal recession, and peri‐implant tissue inflammation in the long term, while a wider KM (≥ 2 mm) appears protective against peri‐implant disease onset. Brushing discomfort declined over time irrespective of KM width.

## 1 Introduction

The peri‐implant mucosa may consist of a keratinized mucosa, which presents a dense connective tissue rich in collagen fibers and firmly adhered to the periosteum (Araujo and Lindhe 2018). A classic study reported that at least 2 mm of keratinized tissue is necessary to maintain periodontal health (Lang and Löe 1972). Based on this, it has been suggested that a similar threshold is necessary for peri‐implant health. While there is strong evidence that plaque accumulation, a history of periodontal disease, and frequency of supportive peri‐implant care (SPIC) are risk factors for the development of peri‐implant diseases (Berglundh et al. 2018; Roccuzzo et al. 2023), the role of keratinized mucosa (KM) in this context remains unclear. Thus, the impact of KM on the onset of peri‐implant diseases has been evaluated.

Most cross‐sectional and short‐term longitudinal studies have reported plaque accumulation and/or tissue inflammation in implants with KM < 2 mm (Adibrad et al. 2009; Bouri et al. 2008; Crespi et al. 2010; Gharpure et al. 2021; Monje and Blasi 2019; Perussolo et al. 2018; Roccuzzo et al. 2016; Schrott et al. 2009; Souza et al. 2016; Ueno et al. 2016). Furthermore, some studies suggest greater mucosal recession (Adibrad et al. 2009; Crespi et al. 2010; Farhoudi and Parsay 2018; Kim et al. 2009; Roccuzzo et al. 2016; Schrott et al. 2009; Zigdon and Machtei 2008), bone loss (Bouri et al. 2008; Kim et al. 2009; Perussolo et al. 2018) and brushing discomfort (Gharpure et al. 2021; Perussolo et al. 2018; Roccuzzo et al. 2016; Souza et al. 2016).

It is suggested that the presence of KM acts as a sensory insulator, protecting peri‐implant tissues against trauma/abrasions and thus providing comfort and enabling adequate oral hygiene (Perussolo et al. 2018; Souza et al. 2016). A cross‐sectional study (Souza et al. 2016) reported higher levels of brushing discomfort in implants with KM < 2 mm, which persisted at the 4‐year follow‐up (Perussolo et al. 2018). However, a recent study found that, although brushing discomfort was present at sites with narrow KM, plaque accumulation remained the primary factor influencing inflammation. The discomfort during brushing itself was also suggested as a potential symptom of peri‐implant disease (Perussolo et al. 2022). Furthermore, studies investigating the impact of KM augmentation around implants demonstrated improvements in tissue inflammation and reduction in plaque accumulation (Tavelli et al. 2021; Thoma et al. 2018).

Concerning peri‐implant diagnosis, the literature is controversial, and long‐term studies are still scarce. Some studies report that implants with inadequate KM appear to be more susceptible to peri‐implant diseases (Canullo et al. 2016; Gharpure et al. 2021; Ramanauskaite et al. 2022; Sanz et al. 2022), while other studies indicate limited evidence for an increased risk of peri‐implantitis (Schwarz et al. 2018). Heitz‐Mayfield and Salvi (2018) suggested that KM presence does not appear to be necessary for peri‐implant mucositis prevention if adequate plaque control is maintained. However, differences in diagnostic criteria between studies make long‐term conclusions challenging.

Although several studies demonstrate the influence of KM on peri‐implant parameters, only a few long‐term prospective studies have investigated this relationship, reporting a greater propensity for plaque accumulation, tissue recession, inflammation, and/or bone loss in implants lacking KM (Mancini et al. 2024; Roccuzzo et al. 2025, 2016). Therefore, most studies are limited and heterogeneous. Thus, the precise influence of KM on peri‐implant tissues remains undetermined (Ravidà et al. 2022; Stefanini et al. 2023). This study aimed to evaluate the effect of narrow (< 2 mm) and wide (≥ 2 mm) KM over 10 years on peri‐implant diagnosis and brushing discomfort.

## 2 Materials and Methods

This is a 10‐year follow‐up study of previously published data (Perussolo et al. 2018; Souza et al. 2016). The present study was approved by the ethics committee for human research at the State University of Maringá, Maringá, Brazil (6.613.448) and was conducted in accordance with the STROBE guidelines and the Declaration of Helsinki. All participants received explanations about the study objectives, risks/benefits, and signed the informed consent form. Patients with at least 1 dental implant were initially evaluated at baseline (T0; *n* = 80 patients, 269 implants) (Souza et al. 2016) and 4 years later (T4; *n* = 54 patients, 202 implants) (Perussolo et al. 2018). Patients were then enrolled in an annual supportive peri‐implant care (SPIC), and after 10 years (T10) from the initial assessment, they were invited to return for reevaluation of clinical and radiographic parameters, as well as brushing discomfort. The inclusion/exclusion criteria have been previously described in detail (Perussolo et al. 2018). In addition to these criteria, patients who did not participate in T4 or who underwent soft tissue grafting around the implants were excluded from the analysis.

Demographic parameters such as age, gender, smoking, and medical/dental history were obtained or updated through a written questionnaire. Moreover, implant location, arch region, loading period, type of restoration, and prosthesis retention were also collected.

### 2.1 Peri‐Implant Clinical and Radiographic Measurements

**FIGURE 1:**
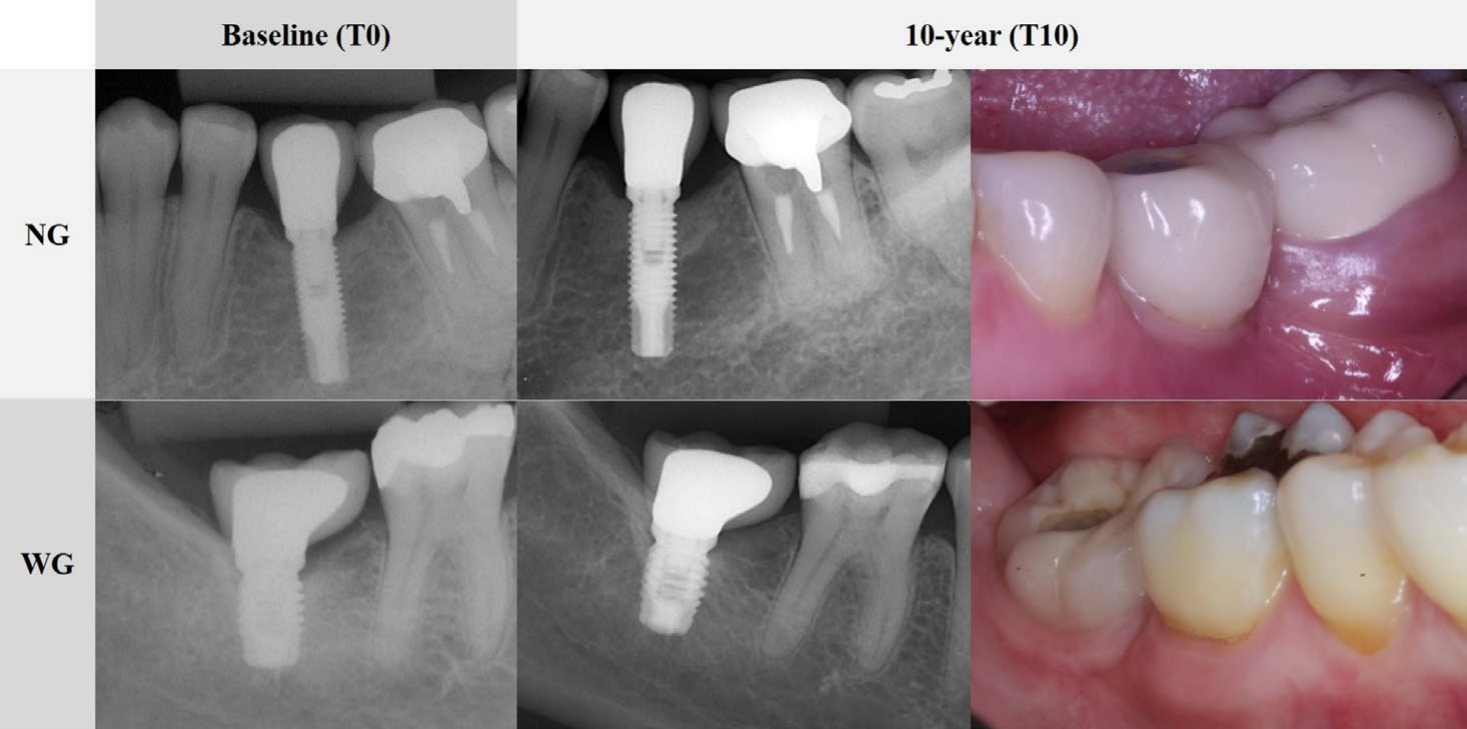
Clinical and radiographic follow‐up of implants in the narrow (NG) and wide (WG) groups over a 10‐year period.

Peri‐implant clinical parameters were assessed at six sites per implant (mesiobuccal, midbuccal, distobuccal, mesiolingual, lingual, and distolingual) using a periodontal probe (Hu‐Friedy, UNC 15, Chicago, USA) by previously calibrated periodontists. The clinical parameters evaluated included plaque index (mPI), probing depth (PD), clinical attachment level (CAL), bleeding on probing (BoP), marginal bone level (MBL), and bone loss (BL). The keratinized mucosa (KM) was assessed only at the midbuccal site. These measurements were detailed in previously published studies (Perussolo et al. 2018; Souza et al. 2016). Additionally, parameters collected but not reported in previous studies were included in the present study, such as mucosal recession (REC) and plaque index (PI—Ainamo and Bay 1975). Implant groups were classified as NG (Narrow group, KM < 2 mm) and WG (Wide group, KM ≥ 2 mm), as shown in Figure 1.

The examiners were previously calibrated to ensure consistency in data collection. Intraobserver error was determined by measuring peri‐implant clinical parameters (PD, CAL, REC, and KM) at 10 dental implants, five in each group of randomly chosen patients. Each measurement was performed twice, with a two‐day interval between assessments. The intraclass correlation coefficient (ICC) was 0.91 (95% CI: 0.83–0.95, *p* < 0.001) and 0.86 (95% CI: 0.74–0.93, *p* < 0.001) for intra‐examiner reliability and inter‐examiner reliability, respectively.

Digital periapical radiographs were obtained for each implant using phosphor plate sensors with the aid of positioning devices utilizing the parallel technique. The captured images were transferred to a computer and analyzed using specific software (Image J, National Institutes of Health, Maryland, USA) calibrated for known measurements, and by a calibrated examiner as described by Perussolo et al. (2018). Bone loss (BL) was calculated by subtracting the MBL at T10 from that at T0.

### 2.2 Peri‐Implant Diagnosis

Diagnosis at the implant level was established according to the new classification proposed by Berglundh et al. (2018), along with the recently suggested updates by Herrera et al. (2023). All implant sites were considered for diagnostic purposes, including clinical measurements and current and previous radiographic evaluations.

- Peri‐implant health was defined as the absence of clinical signs of inflammation, no increase in PD, and no bone loss beyond initial remodeling. Implants presenting with a single BoP spot were also considered healthy.
- Peri‐implant mucositis was diagnosed when BoP was present at more than one site or as line/profuse bleeding, with or without increased PD, and without bone loss beyond initial remodeling.
- Peri‐implantitis was defined by the presence of BoP and/or suppuration, increased PD, and bone loss compared to previous examinations.

### 2.3 Brushing Discomfort

Patients received standardized oral hygiene instructions, including the use of a toothbrush, interdental brushes, and/or dental floss. The recommended brushing technique involved short, vibratory movements at 45° angle, applied for 30 s per site. Immediately after brushing, patients were asked to assess their brushing discomfort (BD) using a visual analog scale (VAS—Jensen et al. 1986). The scale consisted of a 100 mm horizontal line, where patients marked the point that best represented their perceived discomfort. VAS scores were then categorized into presence and absence of BD. The evaluation technique and devices were described in detail in previous studies (Perussolo et al. 2018; Souza et al. 2016).

### 2.4 Spic

Patients were invited to return at least annually for SPIC appointments, during which they were evaluated on clinical and radiographic implant parameters. In cases of peri‐implant mucositis, treatment included mechanical disruption of the biofilm, irrigation with 0.12% chlorhexidine, and reinforcement of oral hygiene instructions and risk‐factor management. In cases of peri‐implantitis, non‐surgical therapy was performed, and the sites were closely monitored. If the condition did not resolve, surgical intervention was recommended (Perussolo and Donos 2024).

### 2.5 Statistical Analysis

Peri‐implant diagnosis was based on six sites per implant. However, only buccal site data were used for peri‐implant parameters comparisons (as per previous publications: Perussolo et al. 2018; Souza et al. 2016). Groups were divided at the implant level according to KM width, and diagnosis was established at both implant and patient level (considering the worst diagnosis within each KM group). Quantitative variables were presented as means and standard deviation (SD) and qualitative variables as frequencies (%). The Shapiro–Wilk test indicated non‐normal distribution for all variables (*p* < 0.001). Group comparisons at each time point were performed using the Mann–Whitney test, while the Friedman test with post hoc Durbin‐Conover analysis was applied for comparisons over time. BD was analyzed by quadrant and categorized as WG (all implants showing KM ≥ 2 mm) or NG (≥ 1 implant with KM < 2 mm). For patients with multiple quadrants within the same group, mean values were calculated. VAS scores were compared between groups using the Mann–Whitney test and described by mean, SD, median, and range. Fisher's exact test was used to compare peri‐implant diagnosis (at both the patient and implant levels) and BD (presence vs. absence) between groups.

To investigate predictors of peri‐implant status, the diagnosis at T10 was modeled as a binary outcome (0 = Health; 1 = Mucositis/Peri‐implantitis). Analyses accounted for the hierarchical structure of the data, with implants nested within patients. Binary implant‐level outcomes at T10 were analyzed using generalized linear mixed‐effects models (GLMMs) with a logit link, including a patient‐level random intercept to account for within‐patient clustering. The multivariable GLMM included all covariates a priori, without automated variable selection. Implant‐ and site‐level covariates comprised group, PI, PD, BoP, and MBL. Patient‐level baseline covariates included sex, diabetes status, history of periodontitis, and SPIC. Continuous variables were standardized (z‐scores) before model fitting. Results were reported as odds ratios (OR) with 95% confidence intervals (95% CIs). The ICC was calculated to quantify between‐patient variability. All analyses were performed in Jamovi (The Jamovi project, 2021, jamovi, Version 1.6, Sydney, Australia) and R (version 4.4.1).

## 3 Results

### 3.1 Sample Description

Of the 80 patients originally enrolled in the study (Souza et al. 2016), 44 returned for the 4‐year evaluation (Perussolo et al. 2018), and 30 patients were included in the 10‐year follow‐up (T10). The reasons for failing to attend T10 were described in Table S1. A total of 30 patients (21 female and 9 male) with a mean age of 61.2 ± 10.2 years participated in the study. Only one patient was a current smoker; two patients had controlled diabetes mellitus, while 10 patients reported a history of periodontal disease, which was stable at the time of assessment. Most patients (*n* = 21) attended the SPIC annually. One hundred and sixteen dental implants with a mean of 15.10 ± 2.15 years of loading period were evaluated at T10. The distribution of implants between maxilla and mandible was similar, while the majority of implants were supporting screw‐retained (95.70%), single crowns (52.60%), in the posterior region (76.70%) of the jaws. Demographic data are shown in Table 1.

**TABLE 1:** Demographic characteristics of patients and implants.

	Patients (*n* = 30)	Implants (*n* = 116)
Age (years)	61.2 ± 10.2	—
Gender		
Male	9 (30%)	—
Female	21 (70%)	—
Smoking history		
Smoker (≤ 10 cigarettes)	1 (3.3%)	—
Ex‐smokers	3 (10%)	—
Never smoked	26 (86.7%)	—
Diabetes mellitus	2 (6.7%)	—
History of periodontal disease	10 (33.3%)	—
Compliant with SPIC	21 (70%)	—
Loading period (years)	—	15.1 ± 2.15
Jaw		
Maxilla	—	57 (49.1%)
Mandible	—	59 (50.9%)
Region		
Anterior	—	27 (23.3%)
Posterior	—	89 (76.7%)
Type of restoration		
Single crowns	—	61 (52.6%)
Fixed partial restorations	—	40 (34.5%)
Full‐arch fixed restorations	—	15 (12.9%)
Retention		
Screw	—	111 (95.7%)
Cemented	—	5 (4.3%)

Abbreviation: SPIC, supportive peri‐implant care.

### 3.2 Clinical and Radiographic Measurements

The mean clinical and radiographic parameters of patients who returned at T10 are presented in Table 2. The width of KM remained stable within groups up to the 10‐year follow‐up; however, KM width was significantly narrower in the NG than in the WG (*p < 0.001*). The mean PI and mPI values remained similar in the WG group, whereas an increase in the amount (mPI) and presence (PI) of biofilm was observed in the NG over the 10 years. A significant increase in PD was identified in both groups at T4, with values remaining stable at T10. In the NG, CAL showed a significant increase over time (*p < 0.001*), whereas in the WG, CAL increased up to 4 years and remained stable in the following years (T4 to T10). No difference was found between groups for PD and CAL at all times. A significant increase in REC was observed in the NG at T10, with NG showing higher REC than WG at all time points. Higher REC was observed in mandibular implants throughout the study (Table S2). In the NG, an increase in BoP was also observed from T0 to T4, with values remaining similar at T10. In contrast, the WG showed a reduction in BoP at the 10‐year follow‐up. Both plaque accumulation (*p < 0.001*) and BoP (*p = 0.007*) at T10 were significantly greater in the NG than in the WG. In the NG, MBL significantly increased at T10, while no significant changes were found in the WG. However, MBL and BL were similar between the groups at all times. Table S3 describes the frequency of PI, mPI, REC, and BoP by site/implant.

**TABLE 2:** Mean and standard deviation (SD) of clinical and radiographic parameters at implant level for NG and WG over time.

		KM (mm)	PI (%)	mPI	PD (mm)	CAL (mm)	REC (mm)	BoP (%)	MBL (mm)	BL (mm)
		Mean ± SD	Mean ± SD	Mean ± SD	Mean ± SD	Mean ± SD	Mean ± SD	Mean ± SD	Mean ± SD	Mean ± SD
NG *n* = 65	T0	0.20 ± 0.40	0.50 ± 0.39^†^	0.63 ± 0.53^†^	2.31 ± 0.60^†^	2.68 ± 0.67^†^	0.36 ± 0.57^†^	0.32 ± 0.38^†^	1.85 ± 0.69^†^	0.44 ± 0.95
	T4	0.18 ± 0.39	0.62 ± 0.41^‡^	0.83 ± 0.64^‡^	2.54 ± 0.66^‡^	2.86 ± 0.66^‡^	0.30 ± 0.43^†^	0.47 ± 0.33^‡^	2.06 ± 0.97^†^	
	T10	0.12 ± 0.33	0.70 ± 0.35^‡^	0.88 ± 0.57^‡^	2.72 ± 0.77^‡^	3.25 ± 1.02^§^	0.52 ± 0.72^‡^	0.48 ± 0.38^‡^	2.30 ± 1.21^‡^	
	*p*	*0.148*	*0.008*	*0.030*	*0.002*	*< 0.001*	*0.002*	*0.002*	*< 0.001*	
WG *n* = 51	T0	3.39 ± 1.80	0.40 ± 0.41	0.52 ± 0.64	2.48 ± 0.69^†^	2.57 ± 0.67^†^	0.09 ± 0.27	0.43 ± 0.39^†^	1.79 ± 0.69	0.19 ± 0.82
	T4	3.23 ± 1.81	0.53 ± 0.41	0.65 ± 0.59	2.79 ± 0.66^‡^	2.90 ± 0.65^‡^	0.11 ± 0.28	0.49 ± 0.36^†^	1.91 ± 0.71	
	T10	3.29 ± 1.87	0.41 ± 0.38	0.45 ± 0.48	2.92 ± 0.75^‡^	3.09 ± 0.80^‡^	0.17 ± 0.44	0.29 ± 0.36^‡^	1.98 ± 1.01	
	*p*	*0.257*	*0.142*	*0.079*	*< 0.001*	*< 0.001*	*0.499*	*0.007*	*0.394*	
NG vs WG	T0	< 0.001	0.178	0.125	0.270	0.384	0.001	0.155	0.683	0.117
	T4	< 0.001	0.217	0.120	0.070	0.832	0.004	0.833	0.632	
	T10	< 0.001	< 0.001	< 0.001	0.138	0.516	< 0.001	0.007	0.119	

*Note:* Mann–Whitney U test was used for intergroup comparisons (NG vs. WG), and the Friedman test with Durbin–Conover post hoc comparisons was applied for intragroup analyses across time. Different symbols indicate differences in the analyzed variable within the same group over the years^†‡§^.

Abbreviations: BL, Bone loss; BoP, Bleeding on Probing; CAL, Clinical attachment level; KM, Keratinized mucosa; MBL, Marginal bone level; mPI, Modified Plaque Index; NG, Narrow group; PD, Probing depth; PI, Plaque Index; REC, mucosal recession; WG, Wide group.

### 3.3 Peri‐Implant Diseases

The prevalence of health, peri‐implant mucositis, and peri‐implantitis at the implant and patient levels over 10 years was shown in Table 3. No significant difference was observed between the studied groups. However, the NG group had a higher number of implants with peri‐implant disease compared to the WG group, with a difference of 15.30% at T0, 13.20% at T4, and increasing to 20% at T10. Notably, the only implant diagnosed with peri‐implantitis at T0 and treated with access flap surgery regressed to mucositis and remained stable for 10 years. At T4, two implants were diagnosed with peri‐implantitis; neither received further treatment, and one was lost and excluded from the current analysis. All implants presenting mucositis at T0 and T4 underwent professional mechanical plaque removal, irrigation with 0.12% chlorhexidine, and received oral hygiene instructions. At the patient level, a higher prevalence of peri‐implant health was observed in the WG at T0 (*p* = 0.009). Although no statistically significant differences were detected over time, patients in the WG consistently exhibited a higher prevalence of peri‐implant health than those in the NG. Additionally, mucositis was the most prevalent diagnosis, irrespective of the group evaluated. Figure S1 summarizes peri‐implant diagnosis according to the type of prosthetic rehabilitation. No statistically significant differences were observed.

**TABLE 3:** Prevalence of peri‐implant diagnosis at implant and patient levels at T0, T4, and T10.

	T0			T4			T10		
Implants	NG	WG	*p*	NG	WG	*p*	NG	WG	*p*
Health	13 (20%)	18 (35.3%)	*0.090*	8 (12.3%)	13 (25.5%)	*0.089*	14 (21.5%)	21 (41.2%)	*0.074*
Mucositis	51 (78.5%)	33 (64.7%)		56 (86.2%)	38 (74.5%)		47 (72.3%)	28 (54.9%)	
Peri‐implantitis	1 (1.5%)	0 (0%)		1 (1.5%)	0 (0%)		4 (6.2%)	2 (3.9%)	
	T0			T4			T10		
Patients	NG	WG	*p*	NG	WG	*p*	NG	WG	*p*
Health	0 (0%)	7 (30.4%)	0.009*	0 (0%)	4 (17.4%)	0.109	2 (9.5%)	7 (30.4%)	0.218
Mucositis	20 (95.2%)	16 (69.6%)		20 (95.2%)	19 (82.6%)		15 (71.4%)	14 (60.9%)	
Peri‐implantitis	1 (4.8%)	0 (0%)		1 (4.8%)	0 (0%)		4 (19%)	2 (8.7%)	

Abbreviations: NG, Narrow group; WG, Wide group.

* *p* < 0.05. Prevalence comparisons were performed using Fisher's exact test. As some patients contributed implants to both groups, patient‐level analyses should be interpreted with caution.

Table 4 presents the peri‐implant diagnostic changes over 10 years. Throughout the follow‐up, a clear pattern emerged: in the NG, most implants (58.40%) remained affected by mucositis from the outset to the end of the study, while 15.30% returned to a healthy condition and 6.20% progressed to peri‐implantitis. Only 6.20% of NG implants maintained health for the entire follow‐up. In contrast, within the WG, 39.20% of implants experienced persistent mucositis, whereas 23.60% recovered. Notably, only one implant with mucositis and one that started out healthy developed peri‐implantitis in this group. Overall, 17.60% of implants in the WG remained healthy throughout the 10 years.

**TABLE 4:** Peri‐implant diagnoses over 10 years at the implant level in NG and WG groups.

NG (*n* = 65 implants)			
Diagnosis at T0	Health at T10	Mucositis at T10	Peri‐implantitis at T10
Health	4 (6.20%)	9 (13.90%)	0 (0%)
Mucositis	10 (15.30%)	37 (58.40%)	4 (6.20%)
Peri‐implantitis	0 (0%)	1 (1.50%)	0 (0%)
WG (*n* = 51 implants)			
Diagnosis at T0	Health at T10	Mucositis at T10	Peri‐implantitis at T10
Health	9 (17.60%)	8 (15.70%)	1 (1.95%)
Mucositis	12 (23.60%)	20 (39.20%)	1 (1.95%)
Peri‐implantitis	0 (0%)	0 (0%)	0 (0%)

Abbreviations: NG, Narrow group; WG, Wide group.

### 3.4 Generalized Linear Mixed Model

The results of the GLMM presented in Table 5 suggest that group and BoP were statistically significant predictors of peri‐implant diagnosis, such that:

- Implants in the WG were associated with significantly lower odds of peri‐implant diseases compared to those in the NG. The calculated OR was 0.16 (95% CI: 0.04–0.60), indicating that the odds of being diagnosed with peri‐implant disease at T10 were 84% lower for implants classified as WG at T0.
- BOP was a significant predictor of diagnosis, with an OR of 3.34 (95% CI: 1.49–7.48). Implants presenting at least one buccal site positive for BOP at T0 showed 3.34 times higher odds of being diagnosed with peri‐implant diseases at T10.

**TABLE 5:** Generalized linear mixed model (GLMM) with logit link for binary implant‐level outcomes at the 10‐year follow‐up.

Variable	Estimate (*β*)	Std. error	*p* value	OR	95% CI
Intercept	1.775	1.004	0.077	5.901	0.826–42.193
Group (wide)	−1.849	0.682	0.007	0.157	0.041–0.600
PI	−0.604	0.340	0.076	0.547	0.281–1.065
PD	0.153	0.336	0.649	1.165	0.603–2.249
BOP	1.207	0.411	0.003	3.342	1.492–7.486
MBL	−0.018	0.341	0.958	0.982	0.503–1.918
Sex (Male)	−0.152	0.994	0.879	0.859	0.122–6.028
Diabetes (Yes)	−1.137	1.973	0.565	0.321	0.007–15.335
Periodontitis (Yes)	1.205	1.067	0.259	3.338	0.413–27.014
SPIC (Regular)	0.049	1.062	0.963	1.050	0.131–8.423

Abbreviations: 95% CI, confidence interval; BoP, bleeding on probing; MBL, marginal bone level; OR, odds ratio; PD, robing depth; PI, plaque index; SPIC, supportive peri‐implant care.

No statistically significant associations were observed for PI, PD, MBL, sex, diabetes, periodontitis, or SPIC in the fully adjusted model. No evidence of multicollinearity was detected among covariates (Table S4). Notably, BoP was the only variable that remained significantly associated with peri‐implant diagnosis in the univariable analyses (Table S5).

### 3.5 Brushing Discomfort

**FIGURE 2:**
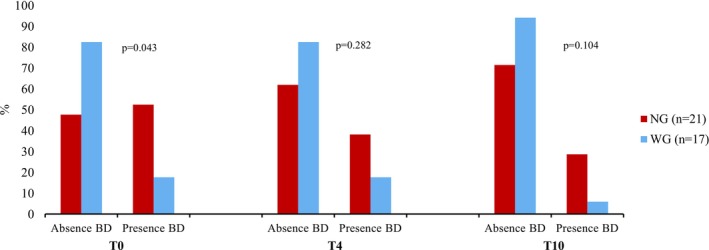
Frequency (%) of brushing discomfort (BD) in the NG and WG at different assessment times.

At T0, the mean VAS score for BD was significantly higher in the NG group (19.10 ± 23.50; median: 0; range: 0–75) compared to the WG group (3.76 ± 10.60; median: 0; range: 0–41), with this difference reaching statistical significance (*p = 0.017*). At T4, the mean VAS was 12.30 ± 19 (median: 0, range: 0–56) for NG and 4.50 ± 14 (median: 0, range: 0–56) for WG. At T10, the mean VAS was 7.00 ± 14.80 (median: 0, range: 0–51) for NG and 0.29 ± 1.21 (median: 0, range: 0–5) for WG. Although no statistically significant differences between groups were found both at T4 (*p = 0.158*) and T10 (*p = 0.062*), BD was more frequently reported in NG quadrants at all periods (T0: 52.40% vs. 17.60%; T4: 38.10% vs. 17.60%; T10: 28.60% vs. 5.90%) with a significant difference between groups observed only at T0 *(p = 0.043)*. All quadrants presenting BD at T10 had at least one implant diagnosed with peri‐implant mucositis, regardless of the KM group. Frequency of BD is illustrated in Figure 2.

## 4 Discussion

The findings of this 10‐year follow‐up study indicate that implants with a narrow KM exhibited greater plaque biofilm accumulation, clinical signs of inflammation, and mucosal recession. Although no direct association between KM and prevalence of peri‐implant diagnosis was identified, implants in the WG showed 84% lower odds of being diagnosed with peri‐implant disease over the years. Furthermore, the presence of BD decreased over the follow‐up period in both groups, and no statistically significant differences were detected at T4 and T10, despite a consistently higher prevalence of discomfort in the NG.

This study found a lower prevalence of peri‐implant diseases compared with previous reports (Galarraga‐Vinueza et al. 2025; Mancini et al. 2024), which may be explained by both the stricter diagnostic criteria adopted for peri‐implant mucositis (Herrera et al. 2023) and the relatively well‐controlled risk profile of the cohort. Most participants were never smokers and non‐diabetic. Only 33% had a history of periodontal disease, and 70% attended SPIC regularly. Long‐term study has shown that the risk of peri‐implant disease increases in the absence of SPIC and in individuals with a history of periodontal disease (Roccuzzo et al. 2023). Current guidelines recommend that all patients with dental implants be enrolled in a SPIC program with frequent and individualized visits to prevent peri‐implant disease (Herrera et al. 2023). In addition, non‐surgical treatment of mucositis should be performed as soon as it is diagnosed, and patients should be monitored to avoid recurrence (Herrera et al. 2023). This approach reflects the management applied to most participants in the present study. Furthermore, all prostheses allow adequate access for oral hygiene, facilitating plaque control. Thus, these factors likely contributed to the low incidence of peri‐implantitis observed in this cohort.

Nevertheless, multilevel logistic regression revealed 84% lower odds of developing peri‐implant disease after 10 years of follow‐up in implants with KM ≥ 2 mm. This finding is consistent with a previous study suggesting a protective effect of KM against peri‐implant diseases (Roccuzzo et al. 2025). Another relevant finding was the low rate of regression to peri‐implant health observed among implants in the NG. Previous studies have reported a higher prevalence of mucositis (Gharpure et al. 2021; Grischke et al. 2019; Kabir et al. 2021) and peri‐implantitis (Ramanauskaite et al. 2022; Roccuzzo et al. 2025) in implants with a narrow KM. In addition, a reduced risk of peri‐implantitis in the presence of KM ≥ 2 has been suggested (Canullo et al. 2016). However, some studies did not find a significant association between peri‐implant diagnosis and KM width (Lim et al. 2019; Oliveira et al. 2023). These conflicting findings may be explained by variability in risk profiles as well as differences in diagnostic criteria between studies (Ravidà et al. 2022; Stefanini et al. 2023). In the present study, current clinical guidelines were applied to minimize overdiagnosis (Herrera et al. 2023). Thus, these observations suggest that KM may influence the long‐term risk of peri‐implant disease development.

Consistent with previous literature, implant sites with KM < 2 mm showed a larger plaque accumulation and/or BoP (Gobbato et al. 2013; Lin et al. 2013; Pranskunas et al. 2016; Ramanauskaite et al. 2022; Roccuzzo et al. 2025; Sabri et al. 2025; Stefanini et al. 2023; Zhang et al. 2025). It has been suggested that the presence of KM around implants may contribute to the formation of a more resilient peri‐implant mucosal seal, characterized by a thicker epithelial barrier and a more stable connective tissue interface. In addition, adequate KM may allow patients to perform oral hygiene more effectively and with less discomfort, thereby promoting regular plaque disruption (Gharpure et al. 2021; Perussolo et al. 2018; Souza et al. 2016). This hypothesis may help explain the higher prevalence of peri‐implant disease reported in implants lacking KM in several studies, given that bacterial plaque is a well‐established risk factor for peri‐implant disease. Importantly, BoP at T0 was associated with higher odds of peri‐implant disease diagnosis at T10. However, the large CI likely reflects the use of a composite diagnosis and the assessment of BoP restricted to the buccal sites, which may increase variability. Although isolated BoP does not represent disease, this longitudinal observation reinforces the relevance of early diagnosis and the critical role of regular SPIC in maintaining tissue health, even in clinically healthy implants.

Marginal tissue stability was poorer in NG implants, as greater REC was observed at all times. This finding is consistent with several studies that observed greater REC in implants with KM < 2 mm (Adibrad et al. 2009; Crespi et al. 2010; Farhoudi and Parsay 2018; Kim et al. 2009; Roccuzzo et al. 2025, 2016; Sabri et al. 2025; Schrott et al. 2009; Zigdon and Machtei 2008). Another relevant finding was that REC was more pronounced in implants placed in the mandible compared with the maxilla, whereas no significant differences were observed between anterior and posterior regions. The increased REC may not be related solely to KM width but also to soft tissue thickness, which can compromise tissue stability, particularly in the presence of inflammation (Tavelli and Barootchi 2025). Similar as observed in the teeth, a thin phenotype appears to be associated with an increased risk of recession (Bienz et al. 2022; Tur and Sarıbaş 2024). However, these parameters were not assessed in the present study, and therefore conclusions regarding their influence should be interpreted with caution.

Interestingly, despite the greater MBL observed in the NG sites after T4, no significant difference was detected between NG and WG. This finding contrasts with long‐term studies reporting lower MBL around implants with adequate KM (Mancini et al. 2024; Roccuzzo et al. 2025). In addition, some cross‐sectional studies have suggested a protective effect of KM on the MBL (Bouri et al. 2008; Kim et al. 2009; Monje and Blasi 2019). Nevertheless, other studies did not find significant differences in bone loss when comparing the width of KM (Adibrad et al. 2009; Crespi et al. 2010; Farhoudi and Parsay 2018; Ladwein et al. 2015; Oliveira et al. 2023; Roccuzzo et al. 2016). The heterogeneity in SPIC protocols and management of peri‐implant diseases across studies may partly explain these conflicting data and may have attenuated bone loss (< 0.5 mm) in the NG in the present cohort. Therefore, further long‐term longitudinal studies are needed to better elucidate the influence of KM on marginal bone levels.

The BD values in the present study were higher at NG than WG sites, but a statistically significant difference was observed only at T0. Some studies have reported greater BD in implants with narrow KM (Gharpure et al. 2021; Monje and Blasi 2019; Roccuzzo et al. 2016), whereas others found no difference between sites with narrow or wide KM (Bonino et al. 2018; Ueno et al. 2016). In a study including patients with peri‐implant health and mucositis, Souza et al. (2016) found significantly higher BD levels at NG than WG sites. Four years later, Perussolo et al. (2018) reevaluated the same sample and confirmed that BD remained higher at NG sites but had decreased over time. In the present study, after 10 years of follow‐up, the difference in BD scores between the experimental groups was further reduced. One possible explanation is that patients who reported higher BD at baseline may have been less motivated to return for the T10 study evaluation, resulting in a sample composed predominantly of individuals with lower initial BD. Furthermore, BD may represent a transient symptom of soft tissue inflammation, which tends to regress with proper oral hygiene maintenance (Perussolo et al. 2022) or through an individual adaptive process over time (Murata and Nakamura 2017). Despite the lack of statistical significance at later time points, BD was consistently more frequent at NG sites suggesting a greater long‐term discomfort associated with narrow KM.

Overall, the results of the present study indicate that WG sites are associated with lower plaque accumulation and inflammation, greater tissue stability, and a lower risk of peri‐implant disease development. Moreover, several studies have demonstrated that an increase in KM promoted better plaque control, less marginal tissue inflammation, recession and/or need for additional treatments (Bouri et al. 2008; Fickl et al. 2021; Oh et al. 2017; Roccuzzo et al. 2025, 2016; Tavelli et al. 2021; Thoma et al. 2018). Based on these findings and current literature, the following clinical approach may be suggested: (i) KM should be evaluated during the initial planning for implant placement and during SPIC to ensure that appropriate preventive and therapeutic measures are implemented, (ii) management of soft tissues before, during, and after implant placement is essential to preserve KM whenever possible, (iii) attention to plaque accumulation and oral hygiene should be reinforced, as plaque remains the primary factor associated with peri‐implant tissue inflammation, (iv) increasing KM may be recommended in cases of persistent inflammation, progressive recession, and/or difficulty in maintaining effective plaque control, with or without associated brushing discomfort (Perussolo and Donos 2024).

The results of this study should be interpreted with caution. The sample size decreased by 62% at T10, representing an important limitation, and it cannot be excluded that participants who returned for follow‐up were more motivated regarding their oral health, which may have influenced the results. In addition, due to the limited number of peri‐implantitis cases, mucositis and peri‐implantitis were analyzed as a combined outcome in the multilevel models; therefore, the reported OR should be interpreted cautiously, as it reflects associations with a composite diagnosis rather than with peri‐implantitis alone. Nevertheless, all patients were rigorously evaluated by calibrated professionals according to the predefined study protocol, and the updated classification of peri‐implant diseases was applied to minimize diagnostic bias. Additional analyses accounting for patient‐level data and risk factors were also performed. This was a single‐center study with a predominance of systemically healthy, non‐smoking patients under regular SPIC, which may limit generalizability. Clinical parameters were reported only for the buccal sites, as KM was measured exclusively at the buccal aspect. Future studies should include both buccal and lingual KM measurements. BD was assessed by quadrants to minimize bias, and mean values were calculated within quadrants of the same group to reduce the impact of individual pain thresholds. BoP criteria evolved over time, limiting the distinction between marginal and profuse bleeding at T0 and T4. Moreover, implants diagnosed with peri‐implant disease were treated as clinically indicated during follow‐up. While this approach was ethically and clinically necessary, it may have influenced the outcomes, as the intervention could have altered the natural progression of the condition. Other variables, such as the thickness of mucosa and vestibular depth, were not evaluated but may be important factors to consider in future studies.

## 5 Conclusion

This long‐term study indicates that (i) a narrow KM was associated with increased plaque accumulation, inflammation, and mucosal recession; (ii) a wider KM correlated with a lower likelihood of onset of peri‐implant disease; and (iii) brushing discomfort may decrease over time, regardless of KM width.

## Author Contributions

**Fernanda Angelio da Costa Deller:** data curation, writing – original draft, investigation, formal analysis, writing – review and editing, visualization. **Jeniffer Perussolo:** data curation, investigation, writing – original draft, writing – review and editing. **Andre Barbisan de Souza:** writing – review and editing, writing – original draft, investigation. **Ricardo Puziol de Oliveira:** formal analysis, writing – original draft, writing – review and editing. **Flávia Matarazzo:** investigation, supervision, project administration, writing – original draft, writing – review and editing. **Mauricio G Araujo:** conceptualization, methodology, supervision, project administration, writing – original draft, writing – review and editing.

## Conflicts of Interest

The authors declare no conflicts of interest.

## Supporting information


**Figure S1:** Peri‐implant diagnosis prevalence by type of restoration at three time points (T0, T4, and T10).

**Table S1:** Reasons for sample size reduction over the 10‐year follow‐up period.

**Table S2:** Comparison of mucosal recession (REC) according to arch region and jaws.

**Table S3:** Frequency of PI, mPI, REC and BoP according to sites and implants of each group (NG and WG).

**Table S4:** Correlation matrix of fixed effects from the generalized linear mixed model with logit link for binary implant‐level outcomes at T10.

**Table S5:** Univariate generalized linear mixed models (GLMMs) with logit link and Holm‐adjusted *p* values for binary implant‐level outcomes at T10.

**Data S1:** STROBE Statement—Checklist of items that should be included in reports of *cohort studies*.

## Data Availability

The data that support the findings of this study are available from the corresponding author upon reasonable request.

## References

[clr70123-bib-0001] Adibrad, M. , M. Shahabuei , and M. Sahabi . 2009. “Significance of the Width of Keratinized Mucosa on the Health Status of the Supporting Tissue Around Implants Supporting Overdentures.” Journal of Oral Implantology 35, no. 5: 232–237. 10.1563/AAID-JOI-D-09-00035.1. 19882819

[clr70123-bib-0002] Ainamo, J. , and I. Bay . 1975. “Problems and Proposals for Recording Gingivitis and Plaque.” International Dental Journal 25, no. 4: 229–235. 1058834

[clr70123-bib-0003] Araujo, M. G. , and J. Lindhe . 2018. “Peri‐Implant Health.” Journal of Clinical Periodontology 45, no. 20: S230–S236. 10.1111/jcpe.12952. 29926494

[clr70123-bib-0004] Berglundh, T. , G. Armitage , M. G. Araujo , et al. 2018. “Peri‐Implant Diseases and Conditions: Consensus Report of Workgroup 4 of the 2017 World Workshop on the Classification of Periodontal and Peri‐Implant Diseases and Conditions.” Journal of Clinical Periodontology 45, no. 20: S286–S291. 10.1111/jcpe.12957. 29926491

[clr70123-bib-0005] Bienz, S. P. , M. Pirc , S. N. Papageorgiou , R. E. Jung , and D. S. Thoma . 2022. “The Influence of Thin as Compared to Thick Peri‐Implant Soft Tissues on Aesthetic Outcomes: A Systematic Review and Meta‐Analysis.” Clinical Oral Implants Research 33, no. 23: 56–71. 10.1111/clr.13789. 35763024 PMC9543651

[clr70123-bib-0006] Bonino, F. , B. Steffensen , Z. Natto , Y. Hur , L. P. Holtzman , and H.‐P. Weber . 2018. “Prospective Study of the Impact of Peri‐Implant Soft Tissue Properties on Patient‐Reported and Clinically Assessed Outcomes.” Journal of Periodontology 89, no. 9: 1025–1032. 10.1002/JPER.18-0031. 29802630

[clr70123-bib-0007] Bouri, A. , N. Bissada , M. S. Al‐Zahrani , F. Faddoul , and I. Nouneh . 2008. “Width of Keratinized Gingiva and the Health Status of the Supporting Tissues Around Dental Implants.” International Journal of Oral & Maxillofacial Implants 23, no. 2: 323–326. 18548930

[clr70123-bib-0008] Canullo, L. , D. Peñarrocha‐Oltra , U. Covani , D. Botticelli , G. Serino , and M. Penarrocha . 2016. “Clinical and Microbiological Findings in Patients With Peri‐Implantitis: A Cross‐Sectional Study.” Clinical Oral Implants Research 27, no. 3: 376–382. 10.1111/clr.12557. 25622536

[clr70123-bib-0009] Crespi, R. , P. Capparè , and E. Gherlone . 2010. “A 4‐Year Evaluation of the Peri‐Implant Parameters of Immediately Loaded Implants Placed in Fresh Extraction Sockets.” Journal of Periodontology 81, no. 11: 1629–1634. 10.1902/jop.2010.100115. 20450368

[clr70123-bib-0010] Farhoudi, I. , and S. Parsay . 2018. “Correlation Between Keratinized Tissue Width and Periodontal Indices Around Implant‐Supported Fixed Partial Dentures.” Journal of Advanced Periodontology & Implant Dentistry 10, no. 1: 24–28. 10.15171/japid.2018.005. 35919771 PMC9327443

[clr70123-bib-0011] Fickl, S. , A. Therese Kröger , T. Dietrich , and M. Kebschull . 2021. “Influence of Soft Tissue Augmentation Procedures Around Dental Implants on Marginal Bone Level Changes‐A Systematic Review.” Clinical Oral Implants Research 32, no. Suppl 21: 108–137. 10.1111/clr.13829. 34642978

[clr70123-bib-0012] Galarraga‐Vinueza, M. E. , S. Pagni , M. Finkelman , T. Schoenbaum , and L. Chambrone . 2025. “Prevalence, Incidence, Systemic, Behavioral, and Patient‐Related Risk Factors and Indicators for Peri‐Implant Diseases: An AO/AAP Systematic Review and Meta‐Analysis.” Journal of Periodontology 96, no. 6: 587–633. 10.1002/JPER.24-0154. 40489307 PMC12273760

[clr70123-bib-0013] Gharpure, A. S. , J. M. Latimer , F. E. Aljofi , J. H. Kahng , and D. M. Daubert . 2021. “Role of Thin Gingival Phenotype and Inadequate Keratinized Mucosa Width (<2 Mm) as Risk Indicators for Peri‐Implantitis and Peri‐Implant Mucositis.” Journal of Periodontology 92, no. 12: 1687–1696. 10.1002/JPER.20-0792. 33856690

[clr70123-bib-0014] Gobbato, L. , G. Avila‐Ortiz , K. Sohrabi , C.‐W. Wang , and N. Karimbux . 2013. “The Effect of Keratinized Mucosa Width on Peri‐Implant Health: A Systematic Review.” International Journal of Oral & Maxillofacial Implants 28, no. 6: 1536–1545. 10.11607/jomi.3244. 24278922

[clr70123-bib-0015] Grischke, J. , A. Karch , A. Wenzlaff , M. M. Foitzik , M. Stiesch , and J. Eberhard . 2019. “Keratinized Mucosa Width Is Associated With Severity of Peri‐Implant Mucositis. A Cross‐Sectional Study.” Clinical Oral Implants Research 30, no. 5: 457–465. 10.1111/clr.13432. 30972785

[clr70123-bib-0016] Heitz‐Mayfield, L. J. A. , and G. E. Salvi . 2018. “Peri‐Implant Mucositis.” Journal of Clinical Periodontology 45, no. 20: S237–S245. 10.1111/jcpe.12953. 29926488

[clr70123-bib-0017] Herrera, D. , T. Berglundh , F. Schwarz , et al. 2023. “Prevention and Treatment of Peri‐Implant Diseases—The EFP S3 Level Clinical Practice Guideline.” Journal of Clinical Periodontology 50, no. S26: 4–76. 10.1111/jcpe.13823. 37271498

[clr70123-bib-0018] Jensen, M. P. , P. Karoly , and S. Braver . 1986. “The Measurement of Clinical Pain Intensity: A Comparison of Six Methods.” Pain 27, no. 1: 117–126. 10.1016/0304-3959(86)90228-9. 3785962

[clr70123-bib-0019] Kabir, L. , M. Stiesch , and J. Grischke . 2021. “The Effect of Keratinized Mucosa on the Severity of Peri‐Implant Mucositis Differs Between Periodontally Healthy Subjects and the General Population: A Cross‐Sectional Study.” Clinical Oral Investigations 25, no. 3: 1183–1193. 10.1007/s00784-020-03422-1. 32607828 PMC7878216

[clr70123-bib-0020] Kim, B.‐S. , Y.‐K. Kim , P.‐Y. Yun , et al. 2009. “Evaluation of Peri‐Implant Tissue Response According to the Presence of Keratinized Mucosa.” Oral Surgery, Oral Medicine, Oral Pathology, Oral Radiology, and Endodontics 107, no. 3: e24–e28. 10.1016/j.tripleo.2008.12.010. 19217009

[clr70123-bib-0021] Ladwein, C. , R. Schmelzeisen , K. Nelson , T. V. Fluegge , and T. Fretwurst . 2015. “Is the Presence of Keratinized Mucosa Associated With Periimplant Tissue Health? A Clinical Cross‐Sectional Analysis.” International Journal of Implant Dentistry 1, no. 1: 11. 10.1186/s40729-015-0009-z. 27747633 PMC5005560

[clr70123-bib-0022] Lang, N. P. , and H. Löe . 1972. “The Relationship Between the Width of Keratinized Gingiva and Gingival Health.” Journal of Periodontology 43, no. 10: 623–627. 10.1902/jop.1972.43.10.623. 4507712

[clr70123-bib-0023] Lim, H.‐C. , D. B. Wiedemeier , C. H. F. Hämmerle , and D. S. Thoma . 2019. “The Amount of Keratinized Mucosa May Not Influence Peri‐Implant Health in Compliant Patients: A Retrospective 5‐Year Analysis.” Journal of Clinical Periodontology 46, no. 3: 354–362. 10.1111/jcpe.13078. 30710371

[clr70123-bib-0024] Lin, G.‐H. , H.‐L. Chan , and H.‐L. Wang . 2013. “The Significance of Keratinized Mucosa on Implant Health: A Systematic Review.” Journal of Periodontology 84, no. 12: 1755–1767. 10.1902/jop.2013.120688. 23451989

[clr70123-bib-0025] Mancini, L. , F. J. Strauss , H.‐C. Lim , et al. 2024. “Impact of Keratinized Mucosa on Implant‐Health Related Parameters: A 10‐Year Prospective Re‐Analysis Study.” Clinical Implant Dentistry and Related Research 26, no. 3: 554–563. 10.1111/cid.13314. 38419210

[clr70123-bib-0026] Monje, A. , and G. Blasi . 2019. “Significance of Keratinized Mucosa/Gingiva on Peri‐Implant and Adjacent Periodontal Conditions in Erratic Maintenance Compliers.” Journal of Periodontology 90, no. 5: 445–453. 10.1002/JPER.18-0471. 30461016

[clr70123-bib-0027] Murata, A. , and T. Nakamura . 2017. “Irrational Behavior in Adaptation: Difference of Adaptation Process to Comfort and Discomfort Stimulus When Presented All Together or Intermittently.” In Advances in Cross‐Cultural Decision Making, edited by S. Schatz and M. Hoffman , 133–142. Springer International Publishing. 10.1007/978-3-319-41636-6_11.

[clr70123-bib-0028] Oh, S.‐L. , R. M. Masri , D. A. Williams , C. Ji , and E. Romberg . 2017. “Free Gingival Grafts for Implants Exhibiting Lack of Keratinized Mucosa: A Prospective Controlled Randomized Clinical Study.” Journal of Clinical Periodontology 44, no. 2: 195–203. 10.1111/jcpe.12660. 27930813

[clr70123-bib-0029] Oliveira, C. A. B. , V. L. Pereira , J. N. Dos Santos , N. S. Araujo , and P. R. Cury . 2023. “Influence of Keratinized Mucosa on Peri‐Implant Diseases: A Retrospective Cohort Study in Humans.” Oral and Maxillofacial Surgery 28, no. 1: 331–336. 10.1007/s10006-023-01144-8. 36847879

[clr70123-bib-0030] Perussolo, J. , and N. Donos . 2024. “Maintenance of Peri‐Implant Health in General Dental Practice.” British Dental Journal 236, no. 10: 781–789. 10.1038/s41415-024-7406-8. 38789755 PMC11126374

[clr70123-bib-0031] Perussolo, J. , F. Matarazzo , D. R. Dias , R. P. Oliveira , and M. G. Araújo . 2022. “The Effect of Brushing Discomfort on Peri‐Implant Health in Sites Exhibiting Inadequate Keratinized Mucosa Width: A Cross‐Sectional Study.” Clinical Oral Implants Research 33, no. 12: 1212–1223. 10.1111/clr.14003. 36181373

[clr70123-bib-0032] Perussolo, J. , A. B. Souza , F. Matarazzo , R. P. Oliveira , and M. G. Araújo . 2018. “Influence of the Keratinized Mucosa on the Stability of Peri‐Implant Tissues and Brushing Discomfort: A 4‐Year Follow‐Up Study.” Clinical Oral Implants Research 29, no. 12: 1177–1185. 10.1111/clr.13381. 30346630

[clr70123-bib-0033] Pranskunas, M. , L. Poskevicius , G. Juodzbalys , R. Kubilius , and R. Jimbo . 2016. “Influence of Peri‐Implant Soft Tissue Condition and Plaque Accumulation on Peri‐Implantitis: A Systematic Review.” Journal of Oral & Maxillofacial Research 7, no. 3: e2. 10.5037/jomr.2016.7302. PMC5100642 27833727

[clr70123-bib-0034] Ramanauskaite, A. , F. Schwarz , and R. Sader . 2022. “Influence of Width of Keratinized Tissue on the Prevalence of Peri‐Implant Diseases: A Systematic Review and Meta‐Analysis.” Clinical Oral Implants Research 33, no. Suppl 23: 8–31. 10.1111/clr.13766. 35763022

[clr70123-bib-0035] Ravidà, A. , C. Arena , M. Tattan , et al. 2022. “The Role of Keratinized Mucosa Width as a Risk Factor for Peri‐Implant Disease: A Systematic Review, Meta‐Analysis, and Trial Sequential Analysis.” Clinical Implant Dentistry and Related Research 24, no. 3: 287–300. 10.1111/cid.13080. 35298862 PMC9311272

[clr70123-bib-0036] Roccuzzo, A. , J.‐C. Imber , A. Stähli , et al. 2025. “Role of Keratinized Mucosa on the Risk of Peri‐Implant Diseases and Soft Tissue Dehiscence in the Posterior Mandible‐A 20‐Year Prospective Cohort Study.” Journal of Periodontal Research 60, no. 12: 1212–1221. 10.1111/jre.70018. 40689718 PMC12881885

[clr70123-bib-0037] Roccuzzo, A. , L. Weigel , C. Marruganti , et al. 2023. “Longitudinal Assessment of Peri‐Implant Diseases in Patients With and Without History of Periodontitis: A 20‐Year Follow‐Up Study.” International Journal of Oral Implantology (Berlin, Germany) 16, no. 3: 211–222. 37767616

[clr70123-bib-0038] Roccuzzo, M. , G. Grasso , and P. Dalmasso . 2016. “Keratinized Mucosa Around Implants in Partially Edentulous Posterior Mandible: 10‐Year Results of a Prospective Comparative Study.” Clinical Oral Implants Research 27, no. 4: 491–496. 10.1111/clr.12563. 25706508

[clr70123-bib-0039] Sabri, H. , L. Tavelli , A. T. Sheikh , et al. 2025. “Significance of Peri‐Implant Keratinised Mucosa on Implant Health: An Umbrella Systematic Review With Evidence Mapping and Quantitative Meta‐Meta‐Analysis.” International Journal of Oral Implantology (Berlin, Germany) 18, no. 1: 13–30. 40047360

[clr70123-bib-0040] Sanz, M. , F. Schwarz , D. Herrera , et al. 2022. “Importance of Keratinized Mucosa Around Dental Implants: Consensus Report of Group 1 of the DGI/SEPA/Osteology Workshop.” Clinical Oral Implants Research 33, no. 23: 47–55. 10.1111/clr.13956. 35763021

[clr70123-bib-0041] Schrott, A. R. , M. Jimenez , J.‐W. Hwang , J. Fiorellini , and H.‐P. Weber . 2009. “Five‐Year Evaluation of the Influence of Keratinized Mucosa on Peri‐Implant Soft‐Tissue Health and Stability Around Implants Supporting Full‐Arch Mandibular Fixed Prostheses.” Clinical Oral Implants Research 20, no. 10: 1170–1177. 10.1111/j.1600-0501.2009.01795.x. 19719741 PMC4928380

[clr70123-bib-0042] Schwarz, F. , J. Derks , A. Monje , and H.‐L. Wang . 2018. “Peri‐Implantitis.” Journal of Clinical Periodontology 45, no. 20: S246–S266. 10.1111/jcpe.12954. 29926484

[clr70123-bib-0043] Souza, A. B. , M. Tormena , F. Matarazzo , and M. G. Araújo . 2016. “The Influence of Peri‐Implant Keratinized Mucosa on Brushing Discomfort and Peri‐Implant Tissue Health.” Clinical Oral Implants Research 27, no. 6: 650–655. 10.1111/clr.12703. 26474541

[clr70123-bib-0044] Stefanini, M. , A. Pispero , M. Del Fabbro , et al. 2023. “The Effect of Keratinized Mucosa on Peri‐Implant Health and Patient‐Reported Outcome Measures: A Systematic Review and Meta‐Analysis.” Applied Sciences 13, no. 15: 8631. 10.3390/app13158631.

[clr70123-bib-0045] Tavelli, L. , and S. Barootchi . 2025. “Prevalence, Incidence, Risk, and Protective Factors for Soft Tissue Dehiscences at Implant Sites in the Absence of Disease: An AO/AAP Systematic Review and Meta‐Regression Analysis.” Journal of Periodontology 96, no. 6: 562–586. 10.1002/JPER.24-0119. 40489305 PMC12273775

[clr70123-bib-0046] Tavelli, L. , S. Barootchi , G. Avila‐Ortiz , I. A. Urban , W. V. Giannobile , and H.‐L. Wang . 2021. “Peri‐Implant Soft Tissue Phenotype Modification and Its Impact on Peri‐Implant Health: A Systematic Review and Network Meta‐Analysis.” Journal of Periodontology 92, no. 1: 21–44. 10.1002/JPER.19-0716. 32710810

[clr70123-bib-0047] Thoma, D. S. , N. Naenni , E. Figuero , et al. 2018. “Effects of Soft Tissue Augmentation Procedures on Peri‐Implant Health or Disease: A Systematic Review and Meta‐Analysis.” Clinical Oral Implants Research 29, no. Suppl 15: 32–49. 10.1111/clr.13114. 29498129

[clr70123-bib-0048] Tur, M. , and E. Sarıbaş . 2024. “Investigation of the Clinical Effects of Peri‐Implant Gingival Morphology on Tissue Health.” Journal of Oral Implantology 49, no. 5: 548–555. 10.1563/aaid-joi-D-23-00084. 37776251

[clr70123-bib-0049] Ueno, D. , T. Nagano , T. Watanabe , S. Shirakawa , A. Yashima , and K. Gomi . 2016. “Effect of the Keratinized Mucosa Width on the Health Status of Periimplant and Contralateral Periodontal Tissues: A Cross‐Sectional Study.” Implant Dentistry 25, no. 6: 796–801. 10.1097/ID.0000000000000483. 27548112

[clr70123-bib-0050] Zhang, Z. , Z. Zhang , P. Wang , Y. Zheng , Z. Wang , and Z. Wang . 2025. “The Relationship Between Adequate Keratinized Mucosa and Peri‐Implant Disease: A Systematic Review and Meta‐Analysis.” BMC Oral Health 25: 345. 10.1186/s12903-025-05680-5. 40050830 PMC11883984

[clr70123-bib-0051] Zigdon, H. , and E. E. Machtei . 2008. “The Dimensions of Keratinized Mucosa Around Implants Affect Clinical and Immunological Parameters.” Clinical Oral Implants Research 19, no. 4: 387–392. 10.1111/j.1600-0501.2007.01492.x. 18266873

