# Supplementary material for: The Role of the Keratinized Mucosa in Peri‐Implant Diseases Onset and Brushing Discomfort: A 10‐Year Follow‐Up
Source: Clin Oral Implants Res. 2026 Mar 29;37(7):785–95. doi: 10.1111/clr.70123 (PMC13340482; doi:10.1111/clr.70123)
Supplement: Supplementary file 1 — Figure S1: Peri‐implant diagnosis prevalence by type of restoration at three time points (T0, T4, and T10). [file CLR-37-785-s002.docx]

**Figure 1S.** Peri-implant diagnosis prevalence by type of restoration at three time points (T0, T4, and T10)
