# Supplementary material for: The Role of the Keratinized Mucosa in Peri‐Implant Diseases Onset and Brushing Discomfort: A 10‐Year Follow‐Up
Source: Clin Oral Implants Res. 2026 Mar 29;37(7):785–95. doi: 10.1111/clr.70123 (PMC13340482; doi:10.1111/clr.70123)
Supplement: Supplementary file 2 — Table S1: Reasons for sample size reduction over the 10‐year follow‐up period. [file CLR-37-785-s004.docx]

**Table 1S.** Reasons for sample size reduction over the 10-year follow-up period

| **Reasons** | **n** |
| --- | --- |
| Changed telephone number/could not be contacted | 18 |
| Chose not to participate in the study | 17 |
| Moved to a different city/state | 5 |
| Passed away | 3 |
| Underwent soft tissue graft | 2 |
| Missing information | 2 |
| Uncontrolled diabetes | 1 |
| Absence in 4-year evaluation | 2 |
