# Supplementary material for: The Role of the Keratinized Mucosa in Peri‐Implant Diseases Onset and Brushing Discomfort: A 10‐Year Follow‐Up
Source: Clin Oral Implants Res. 2026 Mar 29;37(7):785–95. doi: 10.1111/clr.70123 (PMC13340482; doi:10.1111/clr.70123)
Supplement: Supplementary file 3 — Table S2: Comparison of mucosal recession (REC) according to arch region and jaws. [file CLR-37-785-s005.docx]

**Table 2S.** Comparison of mucosal recession (REC) according to arch region and jaws

|  | Anterior *(n=27)*  mean ± SD | Posterior *(n=89)*  mean ± SD | *p* |
| --- | --- | --- | --- |
| T0 | 0.25 ±0.59 | 0.24 ±0.45 | *0.672* |
| T4 | 0.17 ± 0.35 | 0.23 ± 0.39 | *0.393* |
| T10 | 0.56 ± 0.88 | 0.31 ± 0.53 | *0.586* |
|  | Maxilla *(n=57)*  mean ± SD | Mandible *(n=59)*  mean ± SD | *p* |
| T0 | 0.11 ± 0.26 | 0.37 ±0.60 | *0.012* |
| T4 | 0.08 ± 0.20 | 0.35 ± 0.46 | *<0.001* |
| T10 | 0.22 ± 0.48 | 0.50 ± 0.74 | *0.012* |

Comparisons were performed using the Mann–Whitney test (*p* < 0.05)
