# Supplementary material for: The Role of the Keratinized Mucosa in Peri‐Implant Diseases Onset and Brushing Discomfort: A 10‐Year Follow‐Up
Source: Clin Oral Implants Res. 2026 Mar 29;37(7):785–95. doi: 10.1111/clr.70123 (PMC13340482; doi:10.1111/clr.70123)
Supplement: Supplementary file 4 — Table S3: Frequency of PI, mPI, REC and BoP according to sites and implants of each group (NG and WG). [file CLR-37-785-s006.docx]

**Table 3S.** Frequency of PI, mPI, REC and BoP according to sites and implants of each group (NG and WG)

|  | T0 | |  | T10 | |
| --- | --- | --- | --- | --- | --- |
|  | **NG** (n=65)  n (%) | **WG** (n=51)  n (%) |  | **NG** (n=65)  n (%) | **WG** (n=51)  n (%) |
| PI (implant) |  |  |  |  |  |
| - | 18 (27.7%) | 20 (39.2%) |  | 7 (10.8%) | 18 (35.3%) |
| +1 sites | 13 (20%) | 14 (27.5%) |  | 11 (16.9%) | 13 (25.5%) |
| +2 sites | 16 (24.6%) | 3 (5.8%) |  | 14 (21.5%) | 9 (17.7%) |
| +3 sites | 18 (27.7%) | 14 (27.5%) |  | 33 (50.8%) | 11 (21.5%) |
| mPI (worst site/implant) |  |  |  |  |  |
| 0 | 18 (27.7%) | 20 (39.2%) |  | 7 (10.8%) | 18 (35.3%) |
| 1 | 30 (46.2%) | 23 (45.1%) |  | 43 (66.1%) | 31 (60.7%) |
| 2 | 16 (24.6%) | 7 (13.7%) |  | 14 (21.6%) | 1 (2%) |
| 3 | 1 (1.5%) | 1 (2%) |  | 1 (1.5%) | 1 (2%) |
| REC (worst site/implant) |  |  |  |  |  |
| 0 mm | 40 (61.5%) | 45 (88.4%) |  | 29 (44.6%) | 43 (84.4%) |
| 1 mm | 16 (24.6%) | 3 (5.8%) |  | 25 (38.4%) | 4 (7.8%) |
| 2 mm | 6 (9.2%) | 3 (5.8%) |  | 7 (10.8%) | 4 (7.8%) |
| 3 mm | 3 (4.7%) | 0 (0%) |  | 3 (4.7%) | 0 (0%) |
| ≥ 4 mm | 0 (0%) | 0 (0%) |  | 1 (1.5%) | 0 (0%) |
| BoP (implant) |  |  |  |  |  |
| - | 32 (49.2%) | 19 (37.3%) |  | 18 (27.7%) | 24 (51%) |
| + 1 site | 12 (18.5%) | 9 (17.7%) |  | 17 (26.15%) | 13 (25.5%) |
| + 2 sites | 11 (16.9%) | 12 (23.5%) |  | 13 (20%) | 4 (7.8%) |
| + 3 sites | 10 (15.4%) | 11 (21.5%) |  | 17 (26.15%) | 8 (15.7%) |

**Abbreviations:** NG – Narrow group; WG – Wide group; PI – Plaque index; mPI – Modified plaque index; REC – Mucosal recession; BoP – Bleeding on probing
