# Supplementary material for: The Role of the Keratinized Mucosa in Peri‐Implant Diseases Onset and Brushing Discomfort: A 10‐Year Follow‐Up
Source: Clin Oral Implants Res. 2026 Mar 29;37(7):785–95. doi: 10.1111/clr.70123 (PMC13340482; doi:10.1111/clr.70123)
Supplement: Supplementary file 5 — Table S4: Correlation matrix of fixed effects from the generalized linear mixed model with logit link for binary implant‐level outcomes at T10. [file CLR-37-785-s003.docx]

**Table 4S.** Correlation matrix of fixed effects from the generalized linear mixed model with logit link for binary implant-level outcomes at T10

| Variable | Intercept | Group (wide) | PI | PD | BOP | MBL | Sex (Male) | Diabetes (Yes) | Periodontitis (Yes) |
| --- | --- | --- | --- | --- | --- | --- | --- | --- | --- |
| Group (wide) | −0.331 |  |  |  |  |  |  |  |  |
| PI | −0.090 | 0.264 |  |  |  |  |  |  |  |
| PD | −0.063 | −0.025 | 0.076 |  |  |  |  |  |  |
| BOP | 0.166 | −0.367 | −0.396 | −0.337 |  |  |  |  |  |
| MBL | −0.003 | 0.067 | −0.073 | 0.192 | −0.123 |  |  |  |  |
| Sex (Male) | 0.026 | −0.146 | 0.056 | −0.067 | 0.036 | −0.235 |  |  |  |
| Diabetes (Yes) | −0.302 | 0.138 | −0.095 | 0.119 | −0.168 | 0.180 | −0.404 |  |  |
| Periodontitis (Yes) | −0.260 | −0.203 | −0.189 | 0.002 | 0.273 | −0.321 | 0.169 | −0.341 |  |
| SPIC (Regular) | −0.735 | −0.019 | −0.035 | 0.058 | 0.019 | 0.140 | −0.336 | 0.433 | 0.043 |

**Abbreviations:** PI- plaque index; PD – probing depth; BoP – bleeding on probing; MBL – marginal bone level; SPIC – supportive peri-implant care
