# Supplementary material for: The Role of the Keratinized Mucosa in Peri‐Implant Diseases Onset and Brushing Discomfort: A 10‐Year Follow‐Up
Source: Clin Oral Implants Res. 2026 Mar 29;37(7):785–95. doi: 10.1111/clr.70123 (PMC13340482; doi:10.1111/clr.70123)
Supplement: Supplementary file 6 — Table S5: Univariate generalized linear mixed models (GLMMs) with logit link and Holm‐adjusted p values for binary implant‐level outcomes at T10. [file CLR-37-785-s007.docx]

**Table 5S.** Univariate generalized linear mixed models (GLMMs) with logit link and Holm-adjusted *p* values for binary implant-level outcomes at T10

| Variable | OR | 95% CI (Lower) | 95% CI (Upper) | *p* value | Holm-adjusted *p* |
| --- | --- | --- | --- | --- | --- |
| Group (wide) | 0.286 | 0.099 | 0.825 | 0.021 | 0.165 |
| PI | 0.804 | 0.487 | 1.330 | 0.392 | 1.000 |
| PD | 1.350 | 0.819 | 2.240 | 0.237 | 1.000 |
| BoP | 2.360 | 1.300 | 4.270 | 0.005 | 0.042 * |
| MBL | 0.934 | 0.573 | 1.520 | 0.783 | 1.000 |
| Sex (Male) | 0.606 | 0.157 | 2.340 | 0.467 | 1.000 |
| Diabetes (Yes) | 0.881 | 0.085 | 9.130 | 0.916 | 1.000 |
| Periodontitis (Yes) | 1.800 | 0.389 | 8.290 | 0.452 | 1.000 |
| SPIC (Regular) | 0.589 | 0.130 | 2.660 | 0.491 | 1.000 |

**Abbreviations:** PI – plaque index; PD – probing depth; BoP – bleeding on probing; MBL – marginal bone level; SPIC – supportive peri-implant care
